# Application of quasimetagenomics methods to define microbial diversity and subtype *Listeria monocytogenes* in dairy and seafood production facilities

**DOI:** 10.1128/spectrum.01482-23

**Published:** 2023-10-09

**Authors:** Brandon Kocurek, Padmini Ramachandran, Christopher J. Grim, Paul Morin, Laura Howard, Andrea Ottesen, Ruth Timme, Susan R. Leonard, Hugh Rand, Errol Strain, Daniel Tadesse, James B. Pettengill, David W. Lacher, Mark Mammel, Karen G. Jarvis

**Affiliations:** 1 Center for Veterinary Medicine, U.S. Food and Drug Administration, Laurel, Maryland, USA; 2 Center for Food Safety and Applied Nutrition, U.S. Food and Drug Administration, College Park, Maryland, USA; 3 Office of Regulatory Science, Northeast Food and Feed Laboratory, U.S. Food and Drug Administration, Jamaica, New York, USA; 4 Center for Food Safety and Applied Nutrition, U.S. Food and Drug Administration, Laurel, Maryland, USA; University of California Davis, Davis, California, USA

**Keywords:** environmental microbiology, metagenomics, genomics, food-borne pathogens, *Listeria monocytogenes*, microbiome, microbial ecology, seafood and dairy production facilities

## Abstract

Microorganisms frequently colonize surfaces within food production facilities. Detection of *Listeria monocytogenes* in this setting relies on culture-dependent methods, but the complex dynamics of bacterial interactions within these environments and their impact on pathogen detection remain largely unexplored. To address this challenge, we applied both 16S rRNA and shotgun quasimetagenomic (enriched microbiome) sequencing of swab culture enrichments from five seafood and seven dairy production environments. Utilizing 16S rRNA amplicon sequencing, we observed variability between 355 samples taken from these 12 production facilities and a distinctive microbiome for each environment. With shotgun quasimetagenomic sequencing, we were able to assemble *L. monocytogenes* metagenome-assembled genomes (MAGs) from 28 of the 32 culture-positive samples. We compared these MAGs to their corresponding whole-genome sequencing assemblies, which resulted in two polyphyletic clades consisting of *L. monocytogenes* lineages I and II with 13,195 and 25,556 single-nucleotide polymorphism sites, respectively. The remaining four MAGs did not produce sufficient genome coverage. To understand and establish limits for pathogen detection and subtyping using shotgun quasimetagenomics, these same data sets were downsampled *in slilico* to produce a titration series of abundances of *L. monocytogenes* and analyzed. Pathogen detection was achieved for all downsampled data sets, even those with only 3× genome coverage. This study contributes to the understanding of microbial diversity within food production environments and presents insights into the level of genome coverage needed in a metagenome sequencing data set to detect, subtype, and source track a foodborne pathogen

**IMPORTANCE:**

In developed countries, the human diet is predominated by food commodities, which have been manufactured, processed, and stored in a food production facility. Little is known about the application of metagenomic sequencing approaches for detecting foodborne pathogens, such as *L. monocytogenes*, and characterizing microbial diversity in food production ecosystems. In this work, we investigated the utility of 16S rRNA amplicon and quasimetagenomic sequencing for the taxonomic and phylogenetic classification of *Listeria* culture enrichments of environmental swabs collected from dairy and seafood production facilities. We demonstrated that single-nucleotide polymorphism (SNP) analyses of *L. monocytogenes* metagenome-assembled genomes (MAGs) from quasimetagenomic data sets can achieve similar resolution as culture isolate whole-genome sequencing. To further understand the impact of genome coverage on MAG SNP cluster resolution, an *in silico* downsampling approach was employed to reduce the percentage of target pathogen sequence reads, providing an initial estimate of required MAG coverage for subtyping resolution of *L. monocytogenes*.

## INTRODUCTION

Food safety and quality are impacted by the microbial ecology within manufacturing facilities. Industrial surfaces (e.g., utensils, architecture, equipment) within food manufacturing facilities are known sources of microbial food contamination, acting as both vehicles for transmission to food commodities and environmental reservoirs for persistence (1 – 5). Additionally, raw materials, food ingredients, and processing water are known to contribute to the bacterial diversity of the finished product and may also introduce lot-to-lot microbiome variations (1 – 5).

Environmental sampling programs provide proactive and preventative surveillance strategies to identify contamination patterns in food production facilities. These programs aim to protect the food supply and maintain food quality by revealing pathogen presence, persistence, and unsanitary conditions over time. Prior to sampling, each production facility is divided into zones according to the proximity of the sampling area to the food. When collected for FDA inspection and environmental sampling assignments, environmental swab samples are immediately transported to an FDA regulatory field laboratory and analyzed for microbial pathogens, such as *Salmonella*, *Listeria,* and *Escherichia coli*. Pathogen isolates are then processed for whole-genome sequencing (WGS) and uploaded into the GenomeTrakr database where they can be compared to food, clinical, and other environmental swab isolate genomes (6, 7).

Culture-independent metagenomic sequencing technology has been transformative in filling knowledge gaps pertaining to the microbial ecology of clinical, food, and environmental samples. Targeted 16S rRNA amplicon sequencing is a cost-effective workflow that has been used to evaluate the microbiotas in food, meat, and beverage-processing facilities; and their potential to impact food safety and quality; and to demonstrate temporal fluctuations of microbial communities in different types of processing facilities (1, 8 – 10). Some examples include the use of 16S rRNA amplicon sequencing to map microbial ecosystems in breweries (11), determine the bacterial diversity of floor drains in cheese production facilities (12), and examine the contribution of the microbiota of seafood-processing facilities to spoilage and contamination of salmon fillets (13). There have also been studies in domestic built environments that investigated food preparation, such as a study on the microbiota of kitchen surfaces (14).

Shotgun metagenomic sequencing is more informative than targeted 16S rRNA amplicon sequencing since it can provide species-level identification of bacterial communities and their gene content. And with sufficient genome coverage, strain-level identification of foodborne pathogens can be achieved (15 – 17). Shotgun metagenomes can also be used to produce metagenome-assembled genomes (MAGs) of individual taxa within a data set when high levels of genome coverage are achieved (18, 19).

The often-high bacterial burden, and complexity, of intrinsic food microbiomes, and limitations in short read sequencing depth preclude the use of culture-independent workflows, such as metagenomics, for regulatory purposes to detect pathogens in food and environmental samples. However, research laboratories are employing metagenomics to gain insights into the dynamics of culture- dependent foodborne pathogen detection methods. Currently, quasimetagenomic approaches including culture enrichment alone, or in conjunction with techniques that shorten enrichment times, such as multiple displacement amplification and immunomagnetic separation, have been employed to amplify pathogen genomic signatures in foods (20 – 24).

In this study, 16S rRNA amplicon and quasimetagenomic sequencing were performed on environmental swab culture enrichments from dairy and seafood production facilities. The 16S rRNA amplicon data were used to identify differences in the microbiotas within and among the firms. Single-nucleotide polymorphism (SNP) analyses were performed to compare *L. monocytogenes* MAGs from shotgun data sets to their corresponding WGS *L. monocytogenes* genomes from the GenomeTrakr Pathogen detection database. An *in silico* downsampling approach was then employed to reduce the percentage of sequence reads in shotgun metagenomes that varied in their proportional abundances of *L. monocytogenes* to test how a reduction in *L. monocytogenes* sequence reads would impact SNP clustering. Taken together, this study revealed microbiota differences from 12 dairy and seafood facilities and variations in abundances of *L. monocytogenes* subtypes and *Listeria* species that impacted SNP clustering. Additionally, we identified bacteria that co-enrich with *Listeria* in Modified *Listeria* Enrichment Broth (UVM) with antimicrobial supplements, including some that may affect food safety and quality.

## MATERIALS AND METHODS

### Samples used in this study

Our sequence data sets were produced from 355 environmental swab UVM *Listeria monocytogenes* culture enrichments from 7 dairy (245 samples) and 5 seafood (110 samples) production facilities (Table 1) collected from February 2018 to August 2019. After the regulatory culture work was completed, 2 mL aliquots were removed from randomly chosen culture-positive and cuture-negative 48 h UVM culture enrichments for metagenomic DNA extraction. Aliquots were centrifuged at 12,000 × *g* for 10 min, and bacterial pellets were stored at −20°C until DNA extraction was performed. All aliquots for metagenomic analysis were taken after the FDA regulatory assignments were completed when the samples were no longer needed for regulatory analysis and disposition. Aliquots from earlier timepoints of the regulatory workflow, such as un-enriched microbiotas, were not available due to chain of custody policies.

**TABLE 1:** Environmental swab culture enrichments analyzed with 16S rRNA amplicon and shotgun metagenomic sequencing from seven dairy and five seafood manufacturing facilities

Firm	Food type^*a*^	16S RNA^*b*^	Shotgun sequenced^*c*^	%Lm Pos^*d*^	Zone 1^*e*^	Zone 2^*e*^	Zone 3^*e*^
AC	D	12	1	0	NA^*f*^	NA	NA
AM	D	68	7	31	18	21	29
G	D	45	7	44	NA	6	21
I	D	54	7	9	8	15	31
J	D	39	3	36	13	26	0
O	D	18	1	22	2	1	15
V	D	9	1	0	NA	NA	NA
A	S	32	7	13	27	2	3
AS	S	12	1	33	NA	NA	NA
F	S	24	6	67	2	7	15
L	S	29	9	10	13	5	11
W	S	13	2	31	0	5	8

^*a*^ Dairy (D) or seafood (S).

^*b*^ Number of samples analyzed with 16S rRNA amplicon sequencing.

^*c*^ Number of samples analyzed with shotgun metagenomic sequencing.

^*d*^ Percent of 16S rRNA samples culture positive for *Listeria monocytogenes*.

^*e*^ Number of samples collected from each environmental monitoring zone; no zone 4 samples were collected.

^*f*^ NA, zone information not available.

Since FDA procedures for culture-positive samples require whole-genome sequencing of pathogen isolates followed by entry of pathogen genomes into the GenomeTrakr database (NCBI PRJNA215355), the culture results of the dairy and seafood environmental swab enrichments were known prior to metagenomic sequencing (Tables 1 and 2). Of the 355 samples sequenced in this study, 65 diary and 33 seafood samples were culture-positive for *Listeria monocytogenes* (Table 2). Some UVM culture enrichments were also culture-positive for *L. innocua* (21 samples), *L. welshimeri* (1 sample), *L. seeligeri* (1 sample), and *L. ivanovii* (1 sample); these isolates were not sequenced or uploaded into the GenomeTrakr database (Table 2).

**TABLE 2:** Culture enrichment results for environmental swab samples from seven dairy and five seafood manufacturing facilities

Firm	Food type^*a*^	FDA environmental sampling^*b*^	Culture negative^*c*^	*L. monocytogenes* positive	*L. innocua* positive	*L. welshimeri* positive	*L. seeligeri* positive	*L. ivanovii* positve	*Listeria* spp. positive^*d*^
AC	D	64	63	0	0	0	1	0	0
AM	D	68	46	21	1	0	0	0	0
G	D	227	205	21	1	0	0	0	0
I	D	491	474	5	10	0	0	0	2
J	D	208	193	14	0	0	0	1	0
O	D	158	151	4	2	0	0	0	1
V	D	75	70	0	5	0	0	0	0
A	S	212	199	4	0	0	0	0	9
AS	S	70	65	4	0	1	0	0	0
F	S	138	122	16	0	0	0	0	0
L	S	226	221	5	0	0	0	0	0
W	S	158	152	4	2	0	0	0	0

^*a*^ Dairy (D) or seafood (S).

^*b*^ Number of samples collected by the FDA.

^*c*^ Number of samples culture negative for *Listeria* by the regulatory culture-based workflow.

^*d*^ When nonpathogenic *Listeria* species was not confirmed.

### Preparation of genomic DNA

Genomic DNA was extracted from bacterial pellets using either the Qiagen DNeasy Blood and Tissue Kit (Qiagen, Germantown, MD, United States) with modifications (92 bacterial pellets) or the ZymoBIOMICS DNA Miniprep Kit (Zymo, Irvine, CA, United States) (263 bacterial pellets). For samples processed with the DNeasy kit, the Gram-positive protocol was modified to incorporate a pre-lysis step. Bacterial pellets were resuspended with 170 µL of enzymatic lysis solution [20 mM Tris-HCl (pH 8.0), 2 mM sodium EDTA, 1.2% Triton X-100, filter sterilized + 100 mg/mL Lysozyme], incubated for 2 h at 37°C, and then boiled at 95°C for 15 min. After cooling for 5 min, 10 µL of 10% SDS was added.

DNA extraction with the ZymoBIOMICS DNA Miniprep Kit was performed according to the manufacturer’s specifications, using mechanical lysis for 40 continuous minutes with a Vortex Genie 2 (Scientific Industries, Bohemia, NY, United States) and a horizontal adaptor. All samples were stored at −20°C until further processing. Library preparation for 16S rRNA amplicon and shotgun metagenomic sequencing used the same metagenomic DNA.

### 16S rRNA amplicon sequencing method

A two-step amplification protocol similar to the published Illumina protocol (Illumina Inc., San Diego, CA) was employed for 16S rRNA amplicon library preparation. The first amplification step targeted the V1 to V3 hypervariable regions of the 16S rRNA gene with the forward primer 26F (5′ TCG TCG GCA GCG TCA GAT GTG TAT AAG AGA CAG DAG AGT TTG ATC MTG GCT CAG 3′) and reverse primer 534R (5′ GTC TCG TGG GCT CGG AGA TGT GTA TAA GAG ACA GTM TTA CCG CGG CNG CTG GCA C 3′) (Integrated DNA Technologies). PCR products were purified with Agencourt AMPure XP beads (Beckman Coulter, Brea, CA, United States) at 1.25× sample vol and quantified using the Qubit 2.0 fluorometer (Invitrogen, Carlsbad, CA, United States).

The second round of PCR was performed with Illumina Nextera XT indexing primers (Illumina Inc., San Diego, CA) to generate amplicons compatible with Illumina MiSeq sequencing chemistry. Library pools (8pM) were spiked with 10% PhiX Control v3 (8pM) (Illumina Inc., San Diego, CA, USA). The 355 libraries were sequenced with additional libraries from a larger study, in 12 MiSeq runs, multiplexed at 72–96 samples per run in paired-end mode with 325 × 275 cycles using the MiSeq Reagent v3 600-cycle kit (Illumina Inc., San Diego, CA). In every third sequencing run, ZymoBIOMICS community standards were incorporated to assess both extraction and sequencing bias.

### Processing and taxonomic assignment of 16S rRNA gene amplicons

Preprocessing and classification of reads were done as follows. First, we used FLASH (25) to merge paired-end reads keeping read 1 when the reads could not be merged (either due to insufficient overlapping basepairs or poor quality within the overlapping region). We then used the program USEARCH (26) to perform subsequent quality filtering to prepare the 16S rRNA sequence reads for taxonomic classification. This included using the fastx_truncate option to remove the primers, fastq_filter to filter based on a minimum fragment length of 150 bp and ee (expected error) value of 1.0 quality, fastx_uniques to get unique sequences per sample, and unoise3 to check for chimeras. The resulting sequences were then classified using MAPSeq with the default database created using NCBI GenBank and RefSeq sequences annotated as ribosomal RNA with 16S or 18S in the annotation (27).

### Shotgun metagenomic sequencing method

Shotgun metagenomic sequencing libraries were prepared using either the Illumina Nextera XT Library Preparation Kit (39 samples) or the Illumina DNA Prep library Kit (Illumina Inc., San Diego, CA) (13 samples). Libraries were sequenced in 8 MiSeq runs multiplexed at 6–8 samples per sequencing run using MiSeq v3 600-cycle chemistry with 2 × 300 cycles.

### k-mer identification of shotgun metagenomes

Determination of the bacterial composition in shotgun metagenomes from environmental swab culture enrichments was conducted using custom C++ programs developed to compile a k-mer signature database containing multiple unique 30 bp sequences per species, and then each read in the metagenomic fastq file was identified using the 30 bp k-mer probes. For each bacterial species or subspecies, each non-duplicated 30-mer from a reference whole-genome sequence was placed into a database. The k-mers not found in at least two-third of a set of additional genome sequences of the same species, and k-mers found in genomes of other species were removed. The resulting k-mer database used in this work contains 5,900 target entries, each consisting of approximately 40,000 (range 44–80,000) unique k-mers comprising 1,100 different bacterial genera and 3,500 species. Normalization of quasimetagenomic data sets was performed to correct for bias due to differing number of species-specific k-mers used per database entry. The results are tabulated as a percentage of bacterial reads identified (contribution to the microbial population of identified species) for each database entry (github.com/mmammel8/kmer_id). Briefly, after reads from shotgun metagenomic sequence data sets are identified as exact matches to k-mers in the bacterial reference database, the read counts are normalized using the number of species-specific k-mer matches in the database by the following formula: normalized read count = [read count × (attempts/hits)]/(genome length), where hits equal the number of k-mers matched, attempts equal the number of k-mers tested, and genome length is the size of species genomes. For normalization of read counts, all k-mers from all reference genomes were tested for matching k-mers to calculate the database’s coverage.

### Assembly of shotgun reads

Sequence reads from isolated colonies of *Listeria* were assembled *de novo* into contigs using SPAdes (Center for Algorithmic Biotechnology, St. Petersburg State University, St. Petersburg, Russia) genome assembler version 3.13.0 with default parameters. Reads from shotgun metagenomic sequencing were assembled into contigs using SPAdes assembler with the --meta option. *Listeria* contigs were identified by nucleotide BLAST (megablast) of the assembled contigs against reference genomes of *Listeria*. Contigs matching any of ten *L. monocytogenes* reference genomes including serotypes 1/2a, 1/2b, 1/2c, 3a, 4a, and 4b, at a nucleotide identity of at least 88% were retained for further analysis (see Table S1 in the supplemental material).

### SNP alignment of *Listeria* WGS and metagenomic assemblies

To determine the core gene set for SNP alignment, 2,819 annotated genes of *L. monocytogenes* str. 4b F2365 (GenBank accession AE017262.2) were used as references for BLAST analyses against 385 representative whole-genome sequences of *Listeria* species *L. monocytogenes*, *L. innocua*, *L. ivanovii*, *L. marthii*, *L. seeligeri*, and *L. welshimeri*. A gene was excluded if more than one match occurred in a genome or if the gene was absent in more than one of the representative genomes at a 75% sequence identity cutoff. The resulting core backbone contained 1,650 genes covering 1.5 Mbp. Next, the 1,650 genes were aligned by BLAST analysis against the WGS, and metagenome assemblies generated from isolates and samples in this study, respectively. Each assembly’s matching sequence to the reference query was added to an alignment for that gene. A C++ program and a Python program (which are available on GitHub, https://github.com/mmammel8/core_genes) were used to scan each alignment and produce a consensus sequence.

### Phylogenetic analysis

Concatenated backbone SNPs were imported into MEGA 3.1 for phylogenetic analysis (28). Neighbor-joining trees were constructed using the p distance model of nucleotide substitution and pairwise deletion option. The SNP distances are estimated based on the proportion of differences for the pairwise comparisons between samples. The proportions were multiplied by the number of polymorphic backbone sites within each data set (13,195 for *L. monocytogenes* lineage I and 25,556 for *L. monocytogenes* lineage II) and then rounded to the nearest integer to get the estimated number of differences. This is necessary because of gaps (missing data) in the backbone gene alignments from the assemblies.

### *In silico* analysis of *Listeria* in culture-positive shotgun metagenomes

We employed an *in silico* down-sampling process to reduce the total number of sequence reads, in three lineage I culture-positive environmental swab enrichments that varied in proportional abundances of *L. monocytogenes*. The first metagenomic fastq file S105 from dairy firm I, with 5,196,513 sequence reads, (86% proportional abundance of lineage I *L. monocytogenes*) was reduced by downsampling from the original set at a probability of 0.5% to 8% to generate 7 additional fastq files. For all these individual fastq files, the contigs were assembled using SPAdes. The assembled contigs were added to the core gene alignments to determine the SNPs and added to an SNP tree. The same method was followed for MAG S170 from dairy firm G and MAG S064 from dairy firm I with 44% and 0.106% abundances of *L. monocytogenes*, respectively. These samples had reads selected at percent ranges that would provide sufficient numbers of *Listeria* reads for assembly (2%–16% and 86%–66%, respectively).

### Statistics

Following OTU clustering and taxonomic assignments of 16S rRNA amplicon sequences, at the 98% identity threshold to MAPseq hierarchical OTUs, alpha diversity (Observed OTUs) and beta diversity were calculated and assessed using the following R packages: Bioconductor, metagenomeSeq, vegan, phyloseq, and ggplot2 (29). Significance tests were conducted using an ANOVA, ADONIS, and pairwise ADONIS tests. A value of *P* less than 0.05 was considered statistically significant. Beta-diversity was examined using Bray-Curtis dissimilarity matrix. Principal component analysis for Aitchison compositions was used when appropriate, namely, very low diversity samples in which three or fewer taxa were identified. To avoid biased *P*-values and to avoid assumptions on the use of null distribution, both parametric and non-parametric approaches were considered in this study. Alpha diversity metrics were measured using Shannon diversity index with significance determined by the non-parametric Kruskal Wallis tests. False Discovery Rate (FDR) correction was used to correct for multiple hypothesis testing. All statistical analyses were conducted in R Studio (version 1.1.456, using R version 3.6.3) using Vegan package (2.6-2) (30). The visualizations were created using ggplot2 unless otherwise mentioned in the figures.

## RESULTS

### 16S rRNA amplicon sequencing

**Fig 1:**
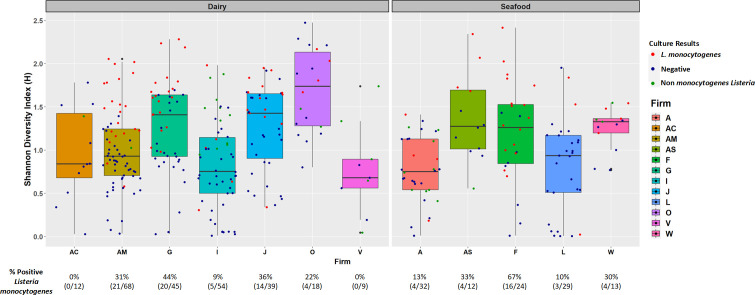
Shannon diversity of enrichments for each sample collected from the seven dairy firms and five seafood firms. Color of the dot indicates the regulatory culture result (blue = *Listeria* culture negative, red = *Listeria monocytogenes* culture positive, green = culture positive for non-monocytogenes *Listeria*). Percentage of *L. monocytogenes* culture-positive samples is indicated at the bottom.

**Fig 2:**
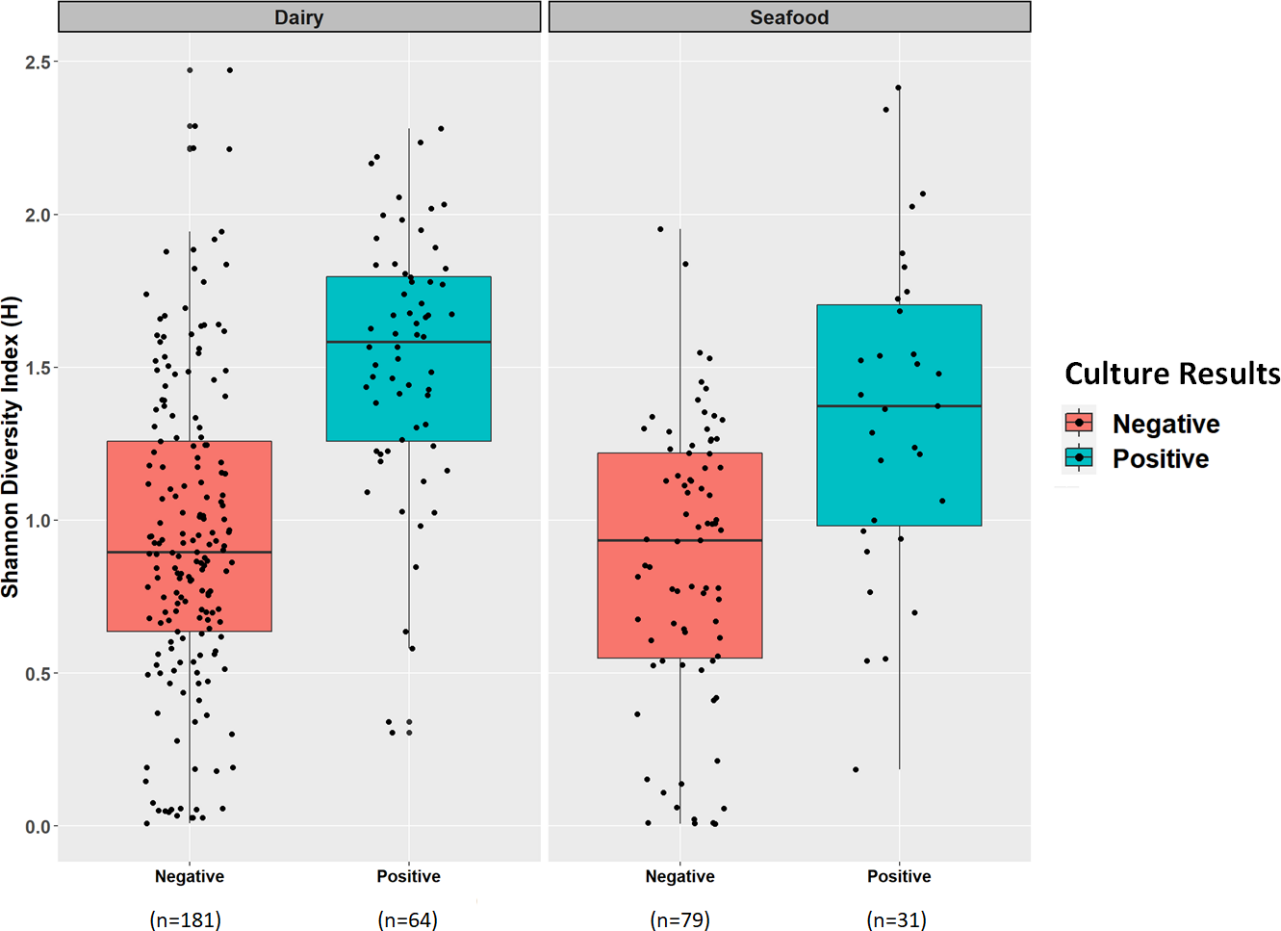
Shannon diversity of 16S rRNA results from 355 environmental swab culture enrichments via box plots. These samples are segregated by the food production facility type (dairy or seafood) and culture results for *L. monocytogenes:* negative dairy (*n* = 181), positive dairy (*n* = 64), negative seafood (*n* = 79), positive seafood (*n* = 31).

**Fig 3:**
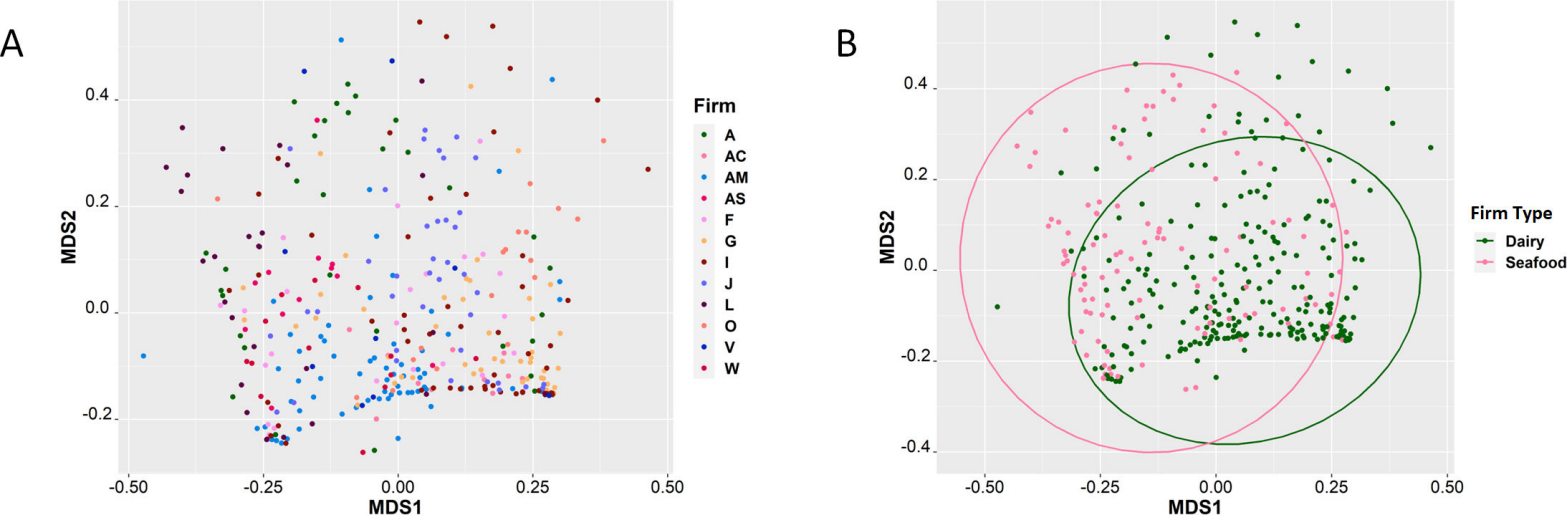
PCA of Bray Curtis dissimilarity distances between the 355 UVM culture enrichments. (**A**) Beta-diversity between samples collected from the 12 food manufacturing firms (AC, AM, G, I, J, O, and V = dairy firms; A, AS, F, L, and W = seafood firms). (**B**) Beta-diversity of samples collected from dairy manufacturing firms (*n* = 245) and seafood manufacturing firms (*n* = 110).

16S rRNA amplicon sequencing of environmental swab culture enrichments from the 12 seafood and dairy manufacturing firms yielded microbiomes that varied in alpha diversity (Fig. 1). However, none of the variation was significant, likely due to the culture bias introduced during enrichment. The enrichment process included antimicrobials which favored the growth of Gram-positive organisms, including *Listeria* species, and had an antagonistic effect against the proliferation of Gram-negative bacteria. Additionally, no significant differences were observed in the Shannon diversity of *L. monocytogenes* culture-positive (*n* = 95) and culture-negative (*n* = 260) enrichment microbiomes (*P* = 0.75) (Fig. 2); however, observationally, the *L. monocytogenes* culture-positive enrichment microbiomes appeared to have higher diversities. Principal component analysis (PCA) of the Bray-Curtis distances did not reveal significant differences (adjusted *P* = 1) in beta diversity among the 245 dairy and 110 seafood culture enrichments from the 12 firms (Fig. 3), nor those that were culture positive or negative for *L. monocytogenes* (see Fig. S1 in the supplemental material).

The potential biases in microbiome diversity introduced by UVM culture enrichment prompted us to test a second beta diversity metric, Aitchison PCA, to account for the possibility of decreased diversity in the enriched microbiomes. The Aitchison PCA metric showed that beta-diversity was driven by a dominance of *Lactococcus*, *Enterococcus,* and *Pseudomonas* for the culture-negative samples and *Listeria* and *Vagococcus* for the culture-positive samples. All of these taxa are within the Gram-positive Firmicutes phyla except *Pseudomonas*.

**Fig 4:**
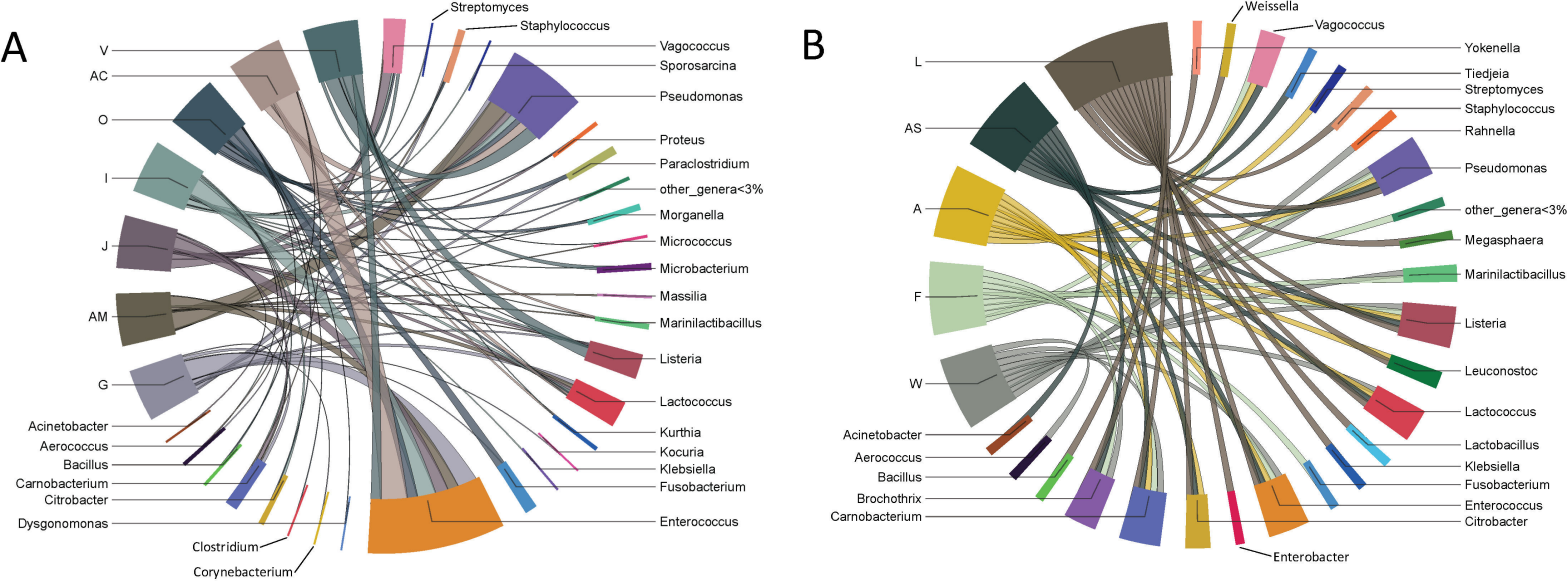
Chord diagrams of shared taxa found in environmental swab culture enrichments taken from seven dairy (**A**) and five seafood (**B**) facilities. All genera less than 3% relative abundance after 16S rRNA amplicon MAPseq analysis have been incorporated into “other_genera <3%.”

The UVM culture enrichment of the environmental swabs from the 12 dairy and seafood firms resulted in bacterial communities dominated by Firmicutes or Proteobacteria (Pseudomonadota) followed by Actinobacteria, Fusobacteria, and Bacteroidetes (Fig. 4; see Table S2 in the supplemental material). As stated above, the Firmicutes population in culture enrichments was dominated by *Enterococcus*, *Lactococcus*, and *Vagococcus*, especially for dairy firms, *Carnobacterium* also predominated in enrichments from some swab samples, mostly from seafood firms (Fig. 4; see Table S2 in the supplemental material). *Pseudomonas* was the dominant taxa in the Proteobacteria phylum in both the dairy and seafood firms (Fig. 4; see Table S2 in the supplemental material).

Variations in proportional abundances of Actinobacteria, Fusobacteria, or Bacteroidetes phyla were observed in some firms. Actinobacteria were observed in 64% (226/355) of all swab culture enrichments. The dairy firms had more samples with Actinobacteria at higher proportional abundances (60%–96% in some samples) than those from seafood firms (see Table S2 in the supplemental material). For example, Actinobacteria was present in 100% of diary firm G samples, which were collected from the firm on three sampling days during 2018 and 2019. The predominant Actinobacteria taxa for each collection day were *Kocuria*, on 4 October 2018; *Rothia* on 4 August 2019; and *Corynebacterium* on 4 August 2019 (see Table S2 in the supplemental material). Fusobacteria 16S rRNA sequence reads were identified in dairy firms O, AM, G, and I and seafood firm F at proportional abundances ranging from 17% to 46% and 12% to 37%, respectively, all classified as *Fusobacterium* (Fig. 4, see Table S2 in the supplemental material). Proportional abundances of Bacteroidetes, namely, *Dysgonomonas*, were low (8% to ≤1%) in both the dairy and seafood firm enrichments (Fig. 4; see Table S2 in the supplemental material).

Differential abundance analyses between the dairy and seafood firms revealed that *Brochothrix*, *Carnobacterium*, *Listeria*, *Leuconostoc*, *Yokenella*, and *Tiedjeia* were significantly higher in dairy firms (*P* < 0.05). Furthermore, *Bacillus*, *Aerococcus*, *Massilia*, *Staphylococcus*, *Proteus*, *Fusobacterium*, *Morganella*, *Paraclostridium*, and *Enterococcus* abundances were significantly lower (*P* < 0.05) in dairy firms compared to seafood firms.

### Shotgun metagenomic analyses

Shotgun metagenomic sequencing of 52 environmental swab culture enrichments provided species-level identification of the microbiomes in a subset of samples from the dairy and seafood firms. *Enterococcus* species were more prevalent in dairy firm culture enrichments and predominated by *E. casseliflavus* (see Table S3 in the supplemental material). K-mer analyses also identified *E. durans*, *E. faecalis*, *E. faecium*, and *E. mundtii. P. putida* and *P. fluorescens* were the most prevalent *Pseudomonas* species in dairy culture enrichments (Sup Table S2). *Carnobacterium maltaromaticum* was present in a few dairy firms at proportional abundances ≤20%, and *Brochothrix thermosphacta* was identified in dairy firm AM microbiotas at proportional abundances ≤4% (see Table S3 in the supplemental material). K-mer analyses of UVM swab enrichments from seafood firms also identified multiple species of *Enterococcus* including *E. casseliflavus*, *E. faecalis,* and *E. aquimarinus*, and *P. fluorescens* was the most predominant *Pseudomonas* species (see Table S3 in the supplemental material). Additionally, the prevalence and proportional abundances of *C. maltaromaticum* and *B. thermosphacta* were higher in seafood firm culture enrichments (see Table S3 in the supplemental material).

### Bacterial fingerprints of taxa in dairy firm AM

**Fig 5:**
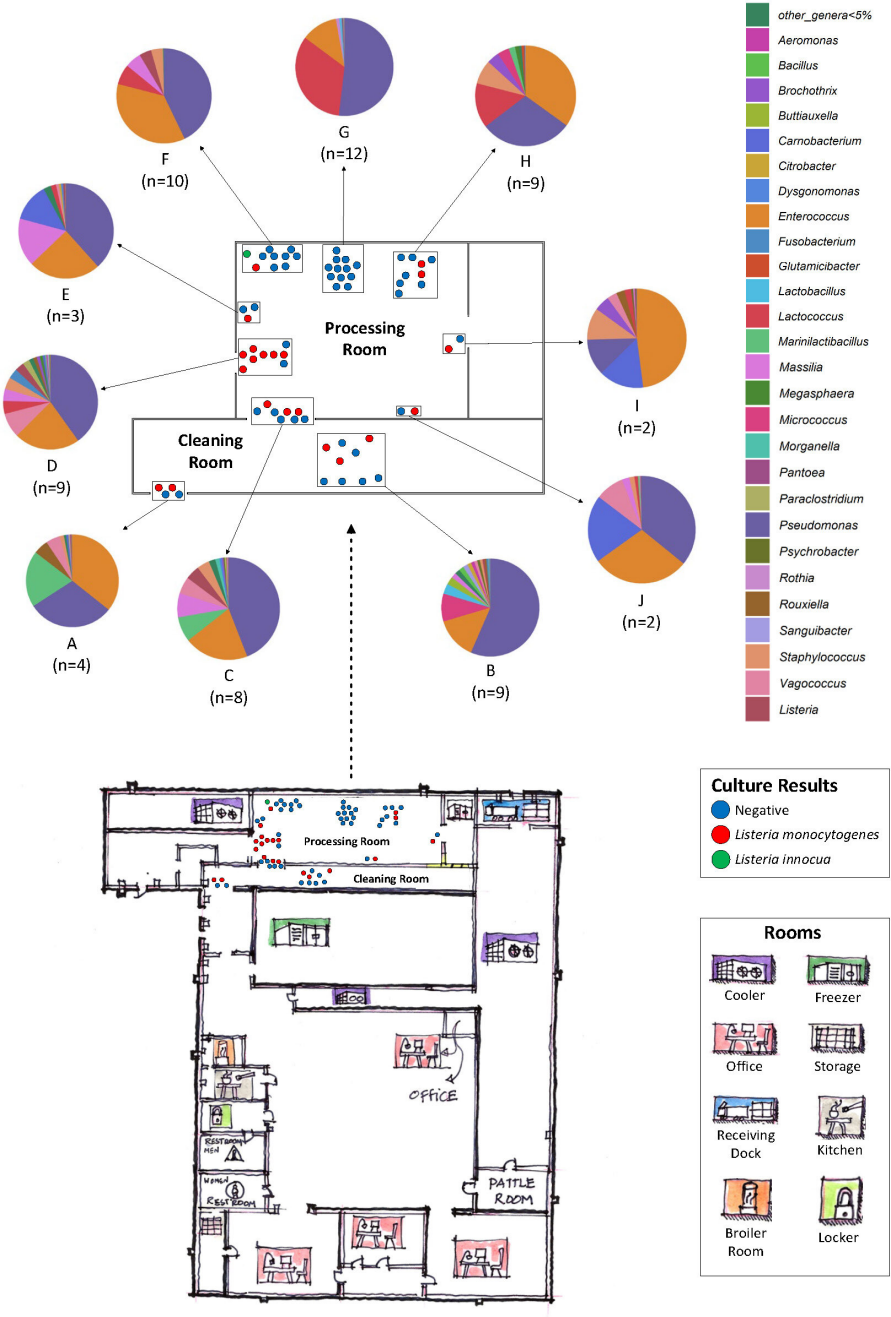
Hypothetical floorplan of dairy production facility FirmAM. Room legend indicates separate rooms of the facility where a specific step in food production occurs. The relative areas sampled (dots) are marked and color coded to reflect the regulatory culture result (blue = negative, red = *Listeria monocytogenes* positive, green = *Listeria innocua* positive). The swab collection sites (*n* = 68) were grouped into 10 sampling areas: Area A (Zone 3), Area B (Zone 2, 3), Area C (Zone 1, 3), Area D (Zone 1, 2, 3), Area E (Zone 3), Area F (Zone 1, 2, 3), Area G (Zone 1, 2), Area H (Zone 1, 2), Area I (Zone 3), and Area J (Zone 3). This floorplan is not drawn to scale.

To determine the potential of employing quasimetagenomics to map the microbiomes in food manufacturing firms, a more thorough analysis of culture enrichments from dairy firm AM, which produces various cheeses and cheese products, was performed. This firm was chosen due to the availability of comprehensive metadata, which was used to create a representative floorplan enabling the identification of the environmental swab enrichment microbiomes in different spatial and environmental monitoring zones within the firm (Fig. 5). Sixty-eight environmental swabs were collected from either the cleaning (*n* = 13) or processing (*n* = 55) rooms and grouped by proximity into 10 areas (A–J) (Table 1; Fig. 5). *L. monocytogenes* was cultured by regulatory analysts from swabs collected in 8 of the 10 areas (Table 1; Fig. 5). Both *L. innocua* and *L. monocytogenes* were cultured from one swab collected in area F, and all swabs from area G were culture negative for *Listeria*.

As was found with the entire quasimetagenomic data set, the 16S rRNA microbiomes of the culture enrichments from the 10 sampling areas from Firm AM were dominated by either *Pseudomonas* (13%–56% proportional abundances) or *Enterococcus* (11%–47% proportional abundances) (Fig. 5). However, lower abundance taxa identified from the cleaning and processing rooms differed. The processing room harbored *Lactococcus* (12%), *Massilia* (4%), and *Staphylococcus* (4%), while the cleaning room areas harbored *Micrococcus* (7%), *Marinilactibacillus* (6%), and *Lactobacillus* (2%) (Fig. 5). Samples from area B of the cleaning room were the most diverse with 23 taxa observed at proportional abundances ≥1% in at least one sample. Area G in the processing room, with the highest number of samples (*n* = 12), harbored the least diverse bacterial communities in enriched samples with only 10 taxa at proportional abundances ≥1% in at least 1 sample; this area also had the highest average proportional abundances of *Lactococcus* (33%); all 12 enrichments were culture negative for *L. monocytogenes*. Additionally, *Carnobacterium* proportional abundances were ≥12% in the processing room areas E, I, and J. *Vagococcus* was observed in all areas except G and H in the processing room, at proportional abundances ranging from 1% to 9% in at least one sample (Fig. 5).

**Fig 6:**
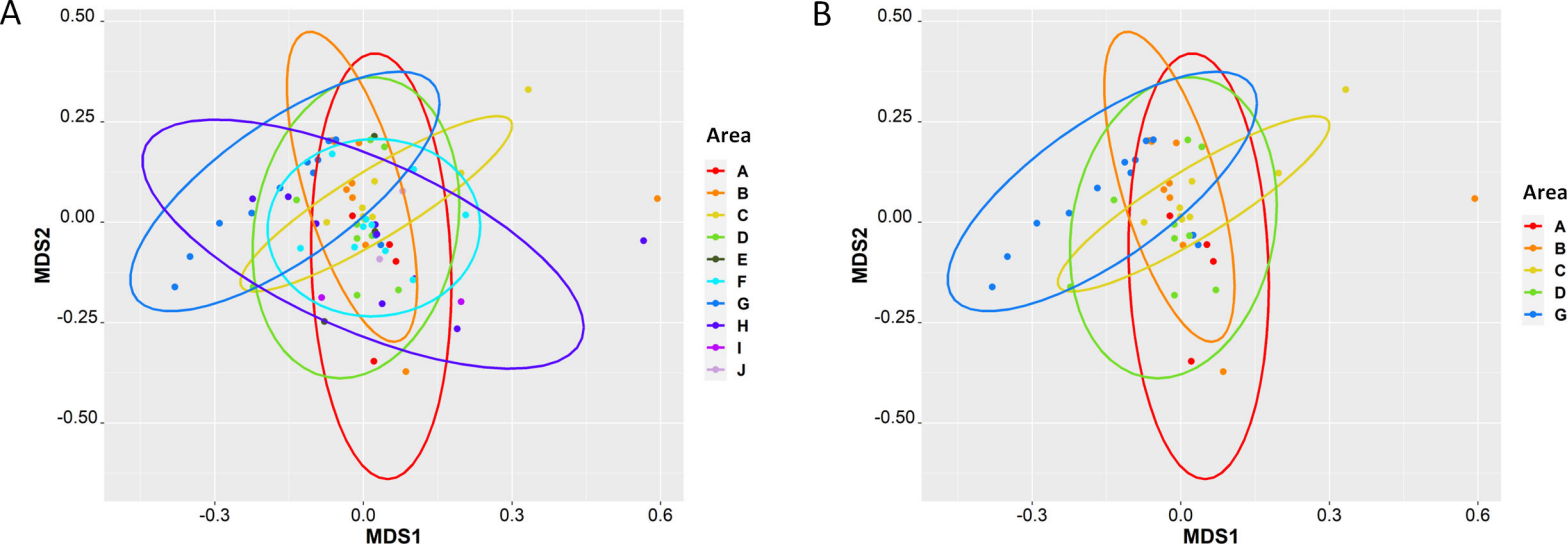
PCA of Bray Curtis dissimilarity distances from culture enrichments from dairy firm AM cleaning and processing rooms. (**A**) Beta-diversity between all samples taken from the 10 areas. (**B**) Areas A (*n* = 4), B (*n* = 9), C (*n* = 8), and G (*n* = 12) had significant differences in beta diversities when compared to the other 6 areas (**D, E, F, H, I, J**).

PCA plots of the Bray-Curtis dissimilarity distances among the microbiomes from firm AM culture enrichments revealed that the beta diversities of area G, in the processing room, significantly differed from swabs collected in areas C (*P* = 0.045) and D (*P* = 0.045), also from the processing room, as well as areas A (*P* = 0.045) and B (*P* = 0.045) from the cleaning room (Fig. 6). These differences were likely due to the high proportional abundances of *Lactococcus* and low diversity of bacteria in area G (Fig. 5). The swabs collected from area G were collected from zone 1 food contact surfaces such as utensils and commonly touched items such as mixing equipment and the outer surfaces of vessels used for processing designated as zone 2. Since firm AM manufactures and processes cheese, it is not surprising that the highest abundances of *Lactococcus* were observed in the processing areas (Fig. 5).

### SNP phylogeny of genomic and metagenomic *Listeria monocytogenes* assemblies

**Fig 7:**
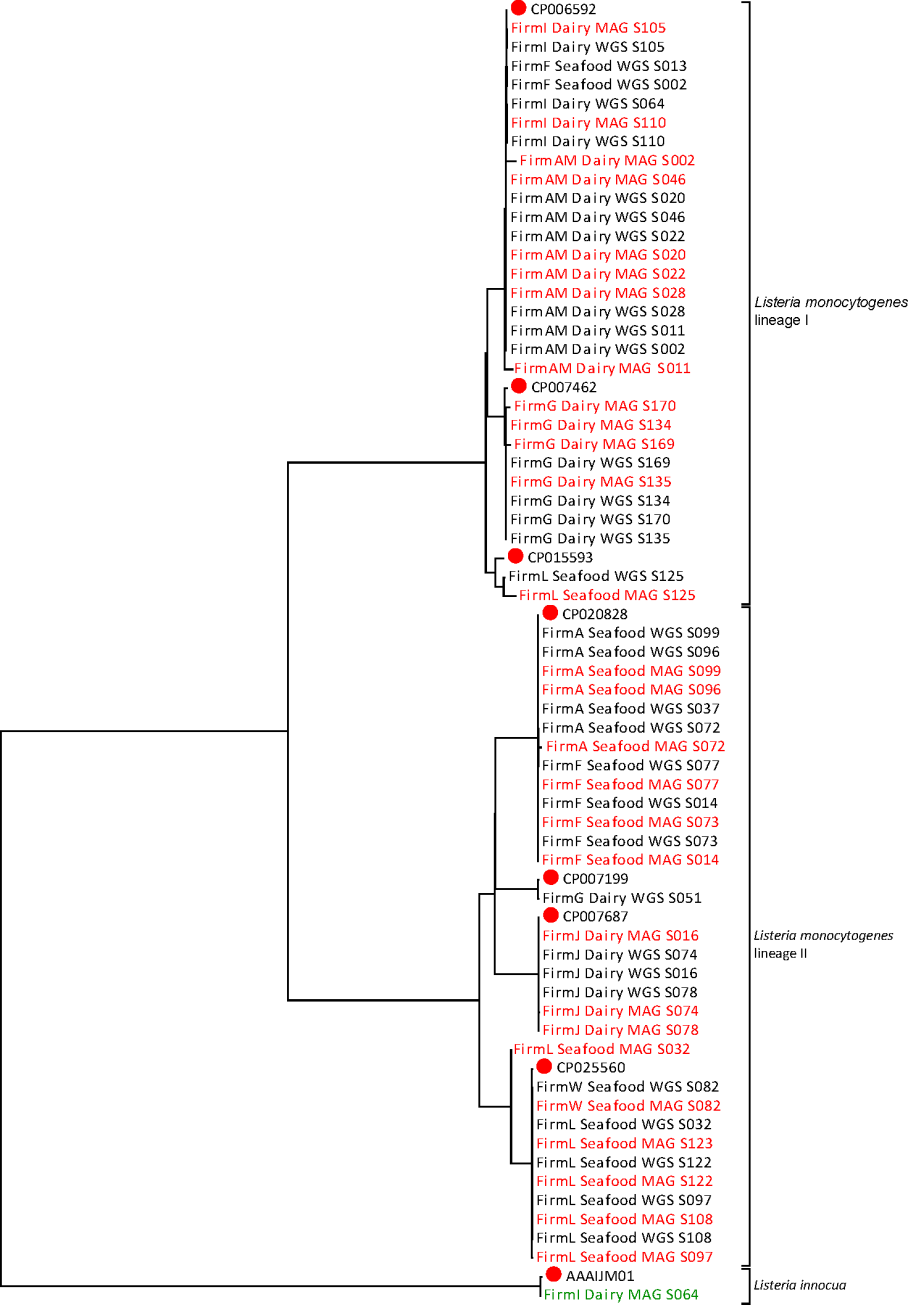
Phylogeny of *Listeria* pure isolates (WGS) and MAG determined by SNP analysis. WGS and MAG are designated by name and color (*Listeria monocytogenes* MAG = red, *Listeria monocytogenes* WGS = black, *Listeria innocua* MAG = green). Samples were named according to the blinded firm name followed by the designation of dairy or seafood manufacturer, whether the sample was sequencing using shotgun metagenomics (MAG) or pure isolate WGS and the environmental swab number. Samples that resolved to *Listeria monocytogenes* lineage I, lineage II, and *Listeria innocua* are included in the tree.

Thirty-two dairy and seafood firm culture-positive swab samples were selected to investigate the concordance of *L. monocytogenes* SNP phylogenies from metagenome-assembled genomes and corresponding WGS of culture isolates, which were available in the GenomeTrakr database. SNP analysis of the *L. monocytogenes* MAGs from 28 of the 32 dairy and seafood swab samples and their corresponding WGS isolate genomes yielded two polyphyletic clades consisting of *L. monocytogenes* lineages I and II with 13,195 and 25,556 SNP sites, respectively (Table S4). The remaining four MAG samples did not produce adequate *L. monocytogenes* genome coverage to be placed in either SNP clade although their corresponding WGS assemblies were placed in the tree (Fig. 7). Both lineages I and II *L. monocytogenes* were cultured from seafood firms F and L and dairy firm G (Fig. 7). Sample S064 from dairy firm I was culture positive for lineage I *L. monocytogenes* and *L. innocua* (Fig. 7). Additionally, the majority of lineage I assemblies are from swabs collected in dairy firms (12/13), while the lineage II clade is mostly from seafood firms (12/15) (Fig. 7).

The lineage I clade of the SNP tree has three subclades consisting of 10 MAGs that group with their corresponding *L. monocytogenes* WGS genome assemblies (Fig. 7; see Fig. S2A in the supplemental material). The CP006592 subclade includes WGS and MAG assemblies from dairy firms AM and I and seafood firm F. The WGS assemblies of dairy firm AM samples S011 and S002 grouped within the CP006592 subclade, but the corresponding MAG assemblies both grouped outside of the clade, likely due to low genome and SNP coverage (Fig. 7; see Fig S2A and Table S4 in the supplemental material). The remaining dairy firm AM WGS and MAG assemblies in the CP006592 subclade are from samples S020, S022, S028, and S046 (Fig. 5 and 7, see Fig. S2 in the supplemental material). Three samples in the CP006592 subclade were collected from dairy firm I. Two of them produced MAG and WGS *L. monocytogenes* assembles that grouped together in this subclade (Fig. 7; see Fig. S2A in the supplemental material). The WGS assembly of S064 from Firm I grouped within the CP006592 subclade, but MAG S064 did not, again likely due to low genome coverage (Fig. 7; see Fig. S2A, Tables S3 and S4 in the supplemental material). Nevertheless, sample S064 was also culture positive for *L. innocua*, and the MAG contained sufficient genome coverage to group with the *L. innocua* reference strain AAAIJM01 (Fig. 7). The final two samples in the CP006592 subclade, S002 and S013, did not have sufficient genome coverage for MAG assemblies, but the WGS isolate genomes were placed in this subclade (Fig. 7; see Fig. S2A and Table S4 in the supplemental material).

**Fig 8:**
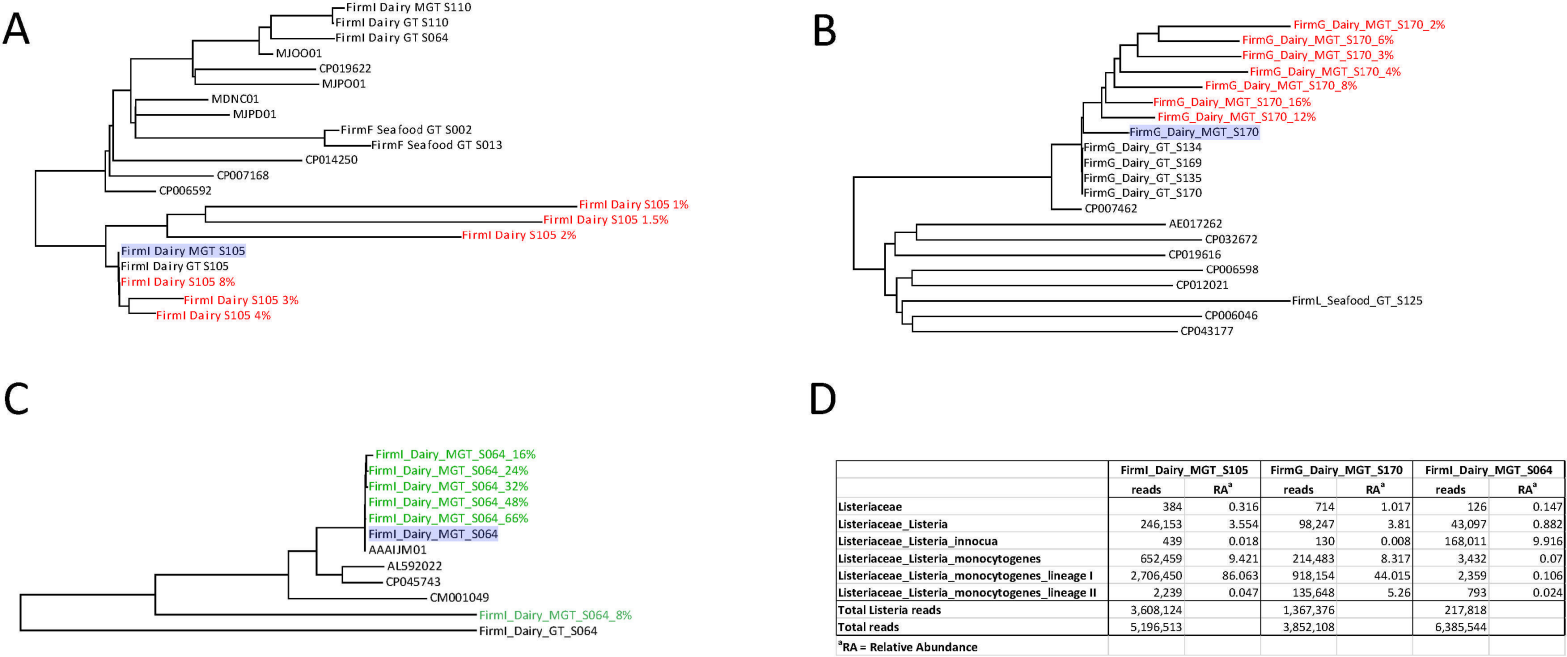
*In silico* fastq dilutions of three metagenomic assembled *Listeria* genomes (**A–C**). The first sample, FirmI_Dairy_MAG_S105 (**A**), had both a high relative abundance of *L. monocytogenes* and a significant number of *L. monocytogenes* lineage I reads. The second sample, FirmG_Dairy_MAG_S170 (**B**), had a high relative abundance and number of *L. monocytogenes* lineage I reads but also exhibited reads for *L. monocytogenes* lineage II. The third and final sample, FirmI_Dairy_MAG_S064 (**C**), was culture positive for both *L. moncytogenes* and *L. innocua,* but shotgun metagenomic sequencing and analysis only identified reads and relative abundance for *L. innocua*. Panel D indicates the sequencing reads and relative abundance data mapped to *Listeria* for all three samples.

A second lineage I SNP subclade comprised of WGS and MAG assemblies from dairy firm G, grouped as subclades of reference strain CP007462. All four WGS assemblies and the MAG assemblies from samples S134 and S135 grouped together in a CP007462 subclade (Fig. 7; see Fig. S2A in the supplemental material). However, MAG assemblies, S169 and S170, had longer branch lengths than their corresponding WGS assemblies (Fig. 7; see Fig. S2A in the supplemental material). The WGS and MAG assemblies for seafood firm L S125 grouped as a subclade of reference genome CP015593; their branch lengths were considerably long due to low SNP coverage (Fig. 8; see Fig. S2A in the supplemental material).

The lineage II *L. monocytogenes* SNP clade is comprised of 13 matching WGS and MAG assemblies within 4 SNP subclades (Fig. 7; see Fig. S2B in the supplemental material). Three samples S014, S073, and S077, collected from seafood firm F produced WGS and corresponding MAG assemblies that grouped with reference strain CP020828 (Fig. 7; see Fig. S2B in the supplemental material). Four samples from seafood firm A grouped with CP020828. MAG S037 from seafood firm A did not group with the corresponding lineage II WGS S037 assembly (Fig. 7; see Table S4 in the supplemental material). The other three MAG assemblies from seafood firm A, S072, S096, and S099, grouped with their corresponding WGS assemblies, as well as the assemblies from firm F in the CP020828 subclade (Fig. 7; see Fig. S2B in the supplemental material).

The lineage II SNP clade also has two subclades with samples collected from dairy firms J and G. The WGS and MAG assemblies from all the samples from these firms grouped in a subclade with reference strain CP007687 (Fig. 7, see Fig. S2B in the supplemental material). The second dairy firm subclade is comprised of reference strain CP007199 and the WGS assembly of firm G, S051 (Fig. 7, see Fig. S2B in the supplemental material). MAG S051 could not be placed in the SNP tree due to low genome coverage (Fig. 7, see Tables S3 and S4 in the supplemental material).

The final lineage II subclade, with reference strain CP025560, is comprised of five samples from seafood firm L and one from seafood firm W (Fig. 7; see Fig. S2B in the supplemental material). Three samples from seafood firm L, S108, S123, and S097, produced WGS and MAG assemblies that grouped together in this subclade (Fig. 7; Fig. S2B in the supplemental material). Seafood firm L MAG assembly S032 placed outside of the CP025560 subclade, while the corresponding WGS assembly is grouped within (Fig. 7; see Fig. S2B in the supplemental material). SNP analysis of the MAG S123, which was culture negative for *L. monocytogenes*, assembled and grouped within the CP025560 lineage II subclade (Fig. 7; see Fig. S2B in the supplemental material). The final sample in the CP025560 subclade is Sample S082 from seafood firm W, which produced WGS and MAG assemblies that grouped in the subclade (Fig. 7; see Fig. S2B in the supplemental material).

### *In silico* analysis of shotgun metagenomes

The SNP analyses of culture-positive quasimetagenomic data sets provided insights into the sequencing depth required to group *L. monocytogenes* MAGs with their corresponding WGS genomes. To take this a step further, we performed *in silico* downsampling on three swab enrichment sample metagenomes to improve our understanding of how reducing, in a step-wise manner, the number of lineage-specific *Listeria* sequence reads in a culture-positive metagenomic enrichment effects SNP phylogeny using this approach.

The first metagenomic data set we analyzed, MAG S105 from dairy firm I, was collected from a zone 3 floor drain in room 1 of the firm. The original MAG S105 contained 2,706,450 *L*. *monocytogenes* sequence reads (86% proportional abundance), which were sequentially downsampled *in silico* to create seven new sequence read data sets, ranging from 0.5% to 8% of the original read set (Fig. 8A and D). The S105 data set with 0.5% of sequence reads was missing all lineage I *L. monocytogenes* SNPs and could not be placed into the S105 SNP tree (Fig. 8A; see Table S5 in the supplemental material). Data sets downsampled to 1%, 1.5%, and 2% of original sequence reads were missing 12,981; 5,381; and 1,471 *L*. *monocytogenes* SNPs, respectively, resulting in genome coverages of 3.4X (0.5%), 3.8X (1.5%), and 4.4X (2%). These three *in silico* downsampled data sets resulted in correct placement in the MAG S105 SNP cluster but had long branch lengths due to reduced genome coverage and missing SNPs (Fig. 8A; see Table S5 in the supplemental material). The S105 MAGs with sequence reads downsampled to 3% and 4%, equivalent to genome coverages of 6.4X and 8.4X, respectively, were placed in a polyphyletic SNP cluster with the original MAG and corresponding WGS S105 genome assembly (Fig. 8A; see Table S5 in the supplemental material). Finally, the read data set downsampled to 8%, which had zero missing SNPs, grouped with the original MAG and the corresponding WGS S105 genome assembly from firm I. The genome coverage for the 8% and original sequence read files were 16.9X (290,711 *L*. *monocytogenes* sequence reads) and 248X (3,623,130 *L*. *monocytogenes* sequence reads), respectively (Fig. 8A; see Table S5 in the supplemental material).

The second *in silico* downsampling was performed with dairy firm G MAG S170 data set, which was from a swab collected in zone 3 on the top of a stair leading to a cheese cave. Dairy firm G MAG S170 had a 44% proportional abundance of lineage I *L. monocytogenes*, equivalent to 918,154 sequence reads, and a 5.3% proportional abundance of lineage II *L. monocytogenes* (135,648 sequence reads) (Fig. 8D; see Table S3 in the supplemental material). *In silico* downsampling of MAG S170 generated sequence data sets with 2%, 3%, 4%, 6%, 8%, 12%, and 16% of total reads. The genome coverage of the original MAG S170 assembly was low (21X with 3,463 missing SNPs) compared to the original dairy firm I MAG assembly S105 (248X with 0 missing SNPs) (see Table S5 in the supplemental material). This low genome coverage resulted in a MAG S170 subclade of the corresponding lineage I WGS S170 assembly and three additional lineage I WGS assemblies from Firm G (Fig. 8B; see Table S5 in the supplemental material). Furthermore, the reduced MAG S170 grouped with the original MAG S170 assembly, with branch lengths that increased as sequence reads were reduced (Fig. 8B). The low genome coverage in MAG S170 is likely due to the presence of lineage II sequence reads, which impact SNP clustering. The additional WGS assemblies, S134, S135, and S169, that grouped with WGS S170 were from swabs collected from the stairs and floor in zone 3 of the cheese cave area in firm G (Fig. 8B; see Table S5 in the supplemental material).

The third set of downsampled MAG data sets was from another firm I sample, S064, collected from the floor under a piece of equipment in room 2 of the firm. The environmental swab culture enrichment for MAG S064 was culture positive for lineage I *L. moncytogenes* and *L. innocua*. Shotgun metagenomic sequencing produced a microbiome with proportional abundances of 10% *L*. *innocua* and <1% lineage I *L. monocytogenes*, equating to 168,011 and 2,359 sequencing reads, respectively (Fig. 8D). *In silico* reduction of MAG S064 produced read data sets with 66%, 48%, 32%, 24% 16%, and 8% total reads, and all except the 8% data set grouped in a SNP cluster with the original MAG S064 sample and AAAIJM01, an *L. innocua* reference isolate; the *L. innocua* isolate from S064 was not sequenced (Fig. 8C). *L. innocua* genome coverages for the fastq files of MAG S064 were 22X (66% fastq), 17X (48% fastq), 13X (32% fastq), 11X (24% fastq), 10X (16% fastq), and 8X (8% fastq), while coverage for the original fastq data set was 31X (Sup Table S4). Both the 8% MAG S064 with 18,853 *Listeria* reads and 275,743 *L*. *innocua* missing SNPs, and the *L. monocytogenes* WGS isolate from sample S064, grouped outside of the *L. innocua* SNP cluster (Fig. 8C and D, see Table S5 in the supplemental material).

## DISCUSSION

Metagenomics is a powerful tool for characterizing microbial communities, and the translation of “omics” technologies like this to food microbiology will have a significant impact in the food industry and for public health (31, 32). The applications of this technology extend far beyond just public health, they can also provide valuable insights about food quality, and there is evidence that the microbiome is likely an important and effective hazard indicator within the food supply chain (33).

We expected to discover significant differences between the enriched microbiomes associated with dairy and seafood food production facilities due to the different food commodities produced in each type of facility. In other studies, it has been reported that food ingredients can act as seeding sources for members of the food production environment microbiome (2, 34). We did not observe any significant differences when looking at the alpha diversity among swabs taken from each firm even though we observed variability between firms and between individual swabs. This is largely due to the sample custody requirement that did not permit access to culture-independent microbiomes in these facilities and is a limitation of this study.

It is well documented that culture enrichment tends to reduce diversity of sample populations as culture conditions, even in non-selective media, tend to favor those organisms that are not fastidious (16, 22, 35, 36). The culture enrichment conditions used in this study, although selective for *Listeria monocytogenes*, allowed growth of other organisms. It is interesting to note that, in general, we did observe increased diversity in swab enrichment microbiomes when *L. monocytogenes* was present. This is due to the fact that in many swab enrichment samples, the relative abundance of *L. monocytogenes* was quite low (<10%), when present, due to co-enrichment of indigenous bacteria. In contrast, in some samples, *Listeria* sp. heavily dominated the quasimetagenome and, thus, lowered alpha diversity.

*L. monocytogenes* is a frequently isolated and sometimes a persistent contaminant within food industry settings (37 – 39). As such, extensive efforts are made to control this pathogen throughout the food supply chain, especially in food production environments, where *Listeria* is commonly transferred to food products by cross contamination from surfaces in production facilities (40, 41). The sanitization and disinfection methods employed to control this, and other pathogens can shape the microbiome and lead to the formation of biofilm communities that may enhance bacterial survival in built food production facilities (39, 41, 42). Additionally, due to the complex architecture and application methods of sanitizers, harborage niches can exist in these built environments.

The dominant taxa observed across all samples were *Enterococcus*, *Pseudomonas*, *Lactococcus*, and *Listeria*. Additionally, *Brochothrix* and *Carnobacterium* were present in swab enrichment samples from seafood facilities as dominant enriched microbiome members. There was a high level of variability in relative abundance from swab-to-swab enrichment microbiomes, even for these dominant taxa, and it was not possible to attribute a certain community or taxa with a facility type, specific zone or sample location, with statistical confidence.

Of note, *Pseudomonas* species were highly abundant in our environmental swab culture enrichments from both dairy and seafood production facilities (Fig. 4 and 5, see Tables S2 and S3 in the supplemental material). While it was not unusual to see high proportional abundances of *Pseudomonas* in foods or food environments, studies have shown that *Pseudomonas* species and *L. monocytogenes* can co-exist in mixed-species biofilms (43 – 47). Additional sampling studies are needed in food production environments to demonstrate that co-existence and mixed species biofilms contribute to food and food facility contamination and harborage.

In addition to the dominant taxa listed above, targeted amplicon sequencing also revealed lower abundant phyla, such as Fusobacteria, Actinobacteria, and Bacteroidetes, that were uniquely enriched in selective UVM cultures from some firms. The identification of *Fusobacterium* 16S rRNA amplicon sequences in 100% of samples from Firm O and 3% to 38% of samples from firms AM, G, I, and F is unusual since this genus consists mostly of obligate anaerobes, except for *F. nucleatum*, which has been demonstrated to survive aerotolerant conditions (48, 49). This finding suggests that anaerobic niches may exist in areas that were sampled in these firms and the subsequent UVM culture enrichment provided a low-oxygen environment allowing *Fusobacterium* to grow. *Dysgonomonas* species, which are known to contribute to polysaccharide utilization in human and insect guts, were the most prevalent Bacteroidetes genus (50, 51). Firm O had the highest number of swab samples with *Dysgonomonas* 16S rRNA sequence reads. Since most of these samples were collected from the floors of the firms, a plausible explanation is a high level of insect activity in these areas of the firms.

We propose that quasi-, and ideally culture-independent, metagenomic analysis in conjunction with a temporal-spatial monitoring plan will provide additional insights into the microbial population structure and dynamics of food production facilities. The significant differences between enriched bacterial communities in the cleaning and processing rooms of dairy firm AM were due to differences in *Lactococcus* proportional abundances, which were higher in area G (33%), compared to areas A (0.09%), B (0.01%), C (0.02%), and D (4%). This is not surprising since area G samples were collected from zone 1 (food contact surfaces) or 2 (the handles and outside of mixing bowls, and commonly touched areas of mixers) where high abundances of *Lactococcus* would be expected in a cheese manufacturing facility. None of the swab samples in area G of firm AM was culture positive for *L. monocytogenes*, and most of the culture-positive swab enrichments (18/21) were collected from zone 3 surfaces such as floors, floor drains, or wheels of moving equipment or carts. Elucidation of bacterial community “fingerprints” in different areas can support efforts to track harborage and transfer patterns of *L. monocytogenes* or other pathogens in this and other production facilities. For example, our observation of the identical lineage II *L. monocytogenes* genomes in MAGs and WGS sequences generated from samples collected 8 months apart in seafood firm L suggests points of harborage in this firm (Fig. 7; see Fig. S2B in the supplemental material). Details about sanitation and disinfection schedules during this time could provide additional data to understand the effects of sanitizers and disinfectants on harborage and persistence facility microbiomes over time.

The availability of *L. monocytogenes* isolate WGS assemblies allowed us to assess and demonstrate the accuracy of SNP clustering of MAGs assembled from the same parent culture enrichments. The comparison of lineages I and II *L. monocytogenes* WGS isolate genomes to corresponding MAG assemblies from parallel quasi-metagenomes revealed disparities in SNP phylogenies of some MAG assemblies (Fig. 7; see Fig. S2 in the supplemental material), making it difficult to determine minimal thresholds for equivalent isolate WGS SNP resolution. Culture-positive samples yielded binomial distributions in terms of *L. monocytogenes* relative abundances, with either high or low levels. In fact, in some samples, insufficient genome coverage precluded the placement of a MAG assembly into the SNP tree.

We estimate that 100,000 sequencing reads mapped to *L. monocytogenes* would be required for consistent cluster resolution; however, this number could be lower under certain conditions (Table S4). Having the WGS assemblies from pure culture isolate provided “ground truth” and allowed us to predict the lineage of the *L. monocytogenes* in each culture enrichment bacterial community. However, the assembly of bacterial genomes from a metagenome is confounded by the presence of sequence reads from other bacterial genomes, especially those that are present in higher abundances. Additionally, metagenomic data sets may have multiple lineages and species of some genera, such as those that are closely related, that reduce the number of unique SNPs, resulting in longer branch lengths due to inadequate genome coverage.

To help us further understand MAG genome coverage impacts on pathogen detection, subtyping, and SNP-level resolution, we performed *in silico* downsampling of selected original quasimetagenomic data sets with varied proportional abundances of *L. monocytogenes*. Pathogen detection was achieved for all downsampled data sets, even those with only 3× genome coverage. Not surprisingly, subtyping resolution increased as the number of *Listeria* reads increased. To summarize, a lineage I subcluster from dairy firm AM had MAG assemblies with 1.1% (S011) and 0.8% (S002) proportional abundances of *L. monocytogenes* that grouped outside of their subclades. However, the corresponding WGS assemblies were grouped within the subclade; samples within the subclade had proportional abundances of *L. monocytogenes* ranging from 2% to 64%.

Among all original and downsampled quasimetagenomic data sets, we observed good subclade assignment with as low as 6× genome coverage. However, this resolution was impacted by the presence of a second *L. monocytogenes* strain or *L. innocua*. For example, *L. innocua* and lineage I *L. monocytogenes* isolates were both cultured from sample S064, but the MAG assembled from this sample suggests that the enrichment process favored *L. innocua*. So, in addition to the identification of differential enrichment dynamics of backbround microbiome members that lead to really high or low relative abundance levels of *L. mononcytogenes*, the presence of multiple strains of *Listeria* and *L. monocytogenes* can negatively impact single strain resolution. The quasimetagenomes generated in this study serve as a foundational data set to quantify and model these interactions. To our knowledge, there are no other studies that address these challenges in the food safety community.

The technology for next-generation sequencing has rapidly evolved over recent years and technical advancement continues in sequencing volume, throughput, and cost reduction. As research labs continue to utilize next-generation sequencing and metagenomic tools as we did here, both culture-independent metagenomic and culture-dependent quasimetagenomic tools have the potential to become a reliable screening tool for analyzing the entire microbial community in environmental swabs and food matrices. This study provides evidence of the utility of quasimetagenomic sequencing methods when used to define microbial diversity in samples taken from different types of food-processing facilities. This study also provides valuable insights into how the metagenomic tools described here could enhance retrospective foodborne outbreak investigations by identifying contamination patterns within food-processing facilities.

## Data Availability

16S rRNA amplicon and shotgun metagenomic sequencing data from this study have been submitted to the food production metagenome BioProject (PRJNA999631). The 16S rRNA amplicon data can be found with accession numbers SAMN36769650 through SAMN36769984. The shotgun metagenomic sequencing data can be found with accession numbers SAMN36768234 through SAMN36768285. The data from this study were submitted using the MIxS food-food production metadata package.

## SUPPLEMENTAL MATERIAL

Supplemental figures and tables spectrum.01482-23-s0001.pdf Fig. S1 and S2; Tables S1, S4, and S5.

Supplemental tables spectrum.01482-23-s0002.xlsx Tables S2 and S3.

ASM does not own the copyrights to Supplemental Material that may be linked to, or accessed through, an article. The authors have granted ASM a non-exclusive, world-wide license to publish the Supplemental Material files. Please contact the corresponding author directly for reuse.
