# Supplementary material for: Application of quasimetagenomics methods to define microbial diversity and subtype Listeria monocytogenes in dairy and seafood production facilities
Source: Microbiol Spectr. 2023 Oct 9;11(6):e01482-23. doi: 10.1128/spectrum.01482-23 (PMC10714831; doi:10.1128/spectrum.01482-23)
Supplement: Supplemental figures and tables — Fig. S1 and S2; Tables S1, S4, and S5. [file spectrum.01482-23-s0001.pdf]

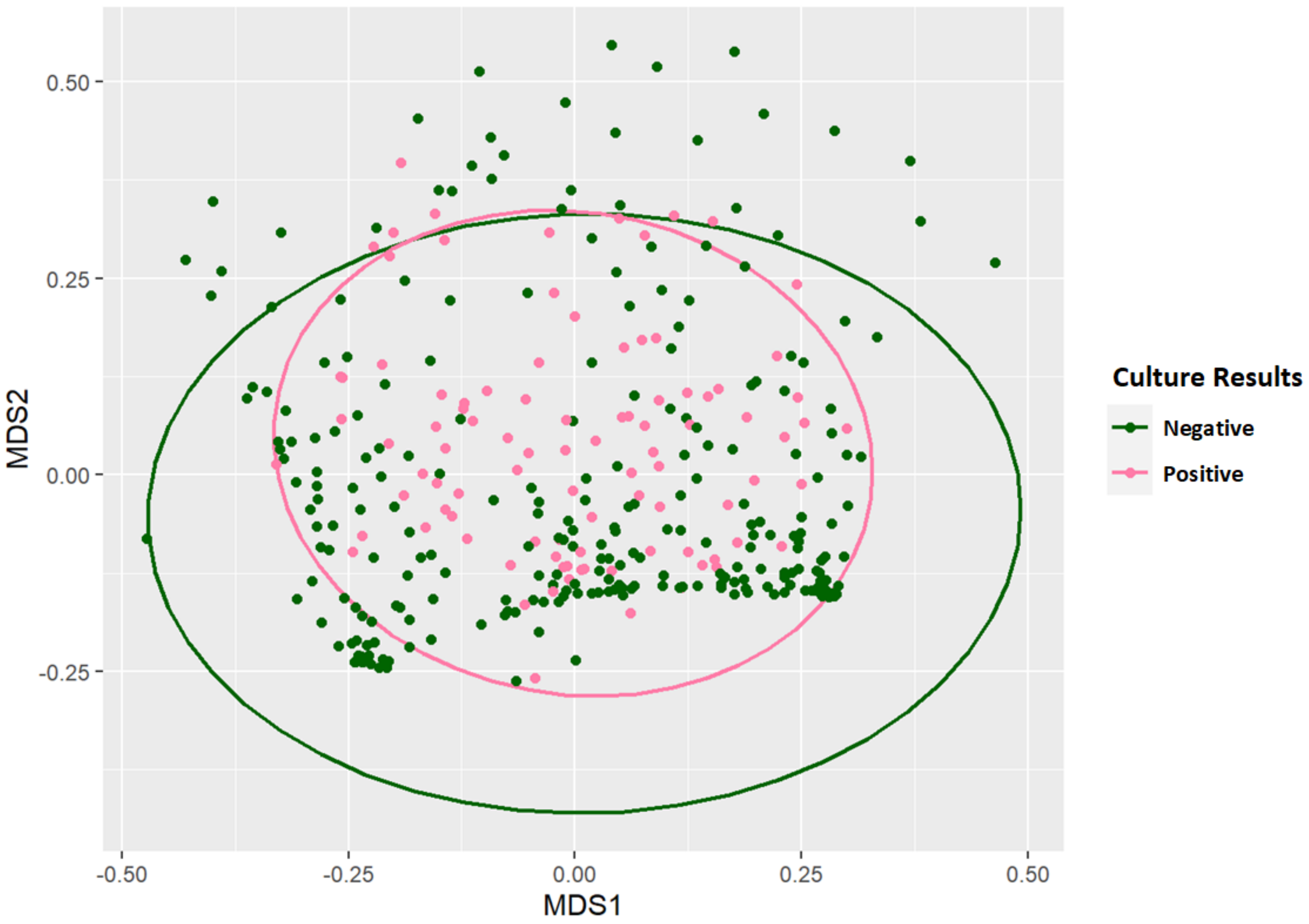

Figure S1: Principal Component Analysis (PCA) of Bray Curtis dissimilarity distances between the 355 UVM culture enrichments. 16S MAPseq data are shown for individual culture enrichments (dots) according to the *Listeria monocytogenes* culture result (designated by color).

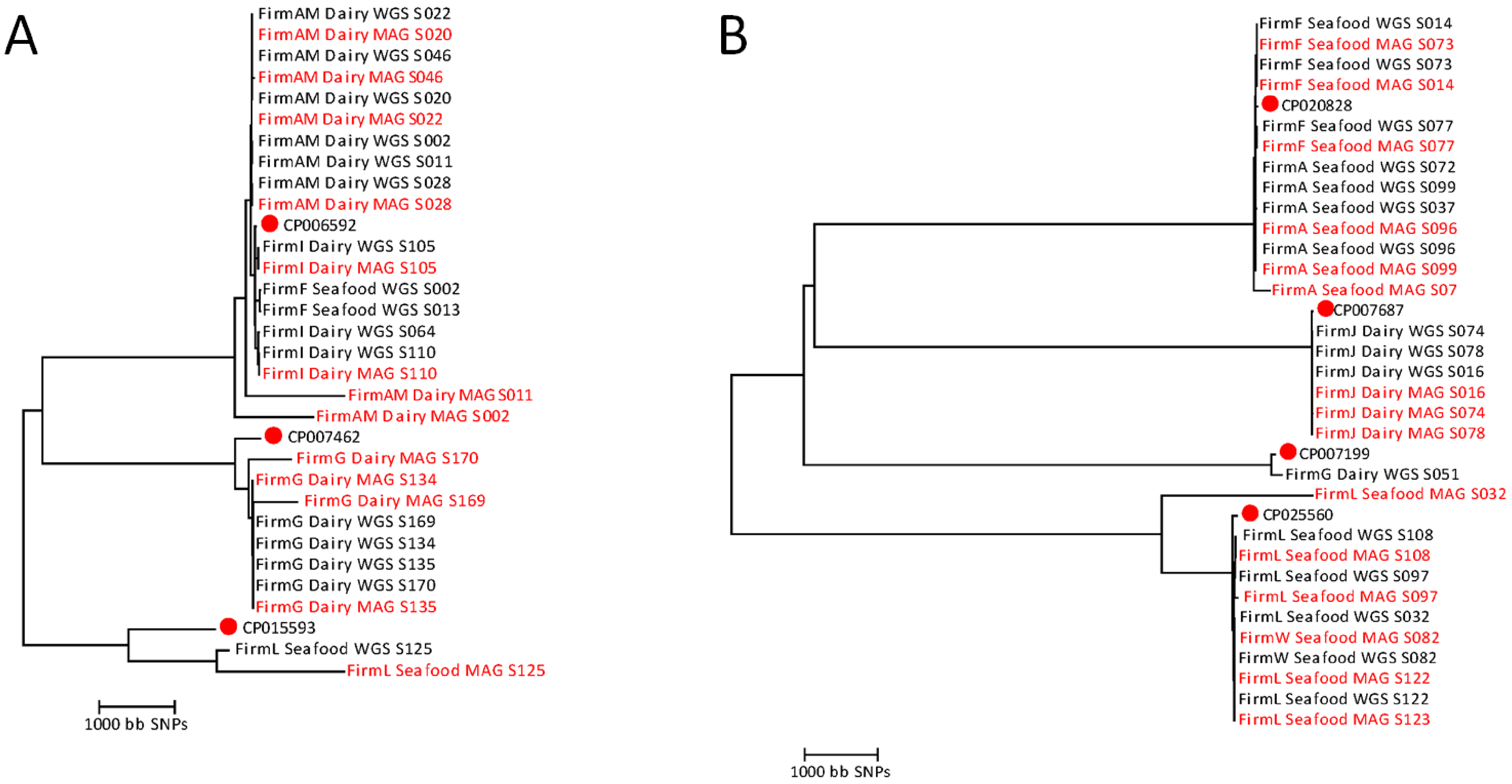

Figure S2: Phylogeny of *Listeria monocytogenes* lineage I (**A**) and *Listeria monocytogenes* lineage II (**B**) determined by single nucleotide polymorphism (SNP) analysis. Refer to **Figure 8** for the entire tree including *Listeria monocytogenes* lineage I and *Listeria monocytogenes* lineage II.

Accession No.	***Listeria monocytogenes* strain**	**Serotype**
CP001602.2	*Listeria monocytogenes* strain 08-5578	serotype 1/2a
CP019623.1	*Listeria monocytogenes* strain 10-092876-1763 LM10	serotype 1/2a
CP007168.1	*Listeria monocytogenes* strain 10-0810	serotype 1/2b
FR733646.1	*Listeria monocytogenes* strain SLCC2755	serotype 1/2b
CP007194.1	*Listeria monocytogenes* strain 10-5025	serotype 1/2c
CP025567.1	*Listeria monocytogenes* strain ATCC 51779	serotype 1/2c
CP002004.1	*Listeria monocytogenes* strain Finland 1998	serotype 3a
CP001175.1	*Listeria monocytogenes* strain HCC23	serotype 4a
AE017262.2	*Listeria monocytogenes* strain F2365	serotype 4b
CP019625.1	*Listeria monocytogenes* strain 10-092876-0769 LM12	serotype 4b

Table S1 *Listeria monocytogenes* reference genomes including serotypes 1/2a, 1/2b, 1/2c, 3a, 4a, and 4b.

Sample Name	Missing SNPs[a]	Reads LM[b]	Reads LM I[c]	Reads LM II[d]	RA LM (%)[h]	Zone	Location
FirmA_Seafood_MAG_S037[f]	164418	871	0	4433	0.252	Zone 1	Utensil
FirmA_Seafood_MAG_S072	12758	31433	77	121552	5.235	Zone 2	Equipment
FirmA_Seafood_MAG_S096	306	700890	1350	2804375	91.89	Zone 1	Equipment
FirmA_Seafood_MAG_S099	306	513971	1119	2047563	75.37	Zone 1	Equipment
FirmAM_Dairy_MAG_S002[e]	103911	5259	28015	14	0.805	Zone 3	Architecture
FirmAM_Dairy_MAG_S011[e]	117617	4345	23047	8	1.1	Zone 2	Equipment
FirmAM_Dairy_MAG_S020	45	429048	2344108	1068	64.23	Zone 3	Equipment
FirmAM_Dairy_MAG_S022	33	101172	549613	295	22.48	Zone 3	Architecture
FirmAM_Dairy_MAG_S028	20	352918	1832153	940	57.63	Zone 3	Equipment
FirmAM_Dairy_MAG_S046	1956	19765	106361	64	1.77	Zone 2	Equipment
FirmF_Seafood_MAG_S002[f]	167214	116	128	289	0.05	Zone 3	Architecture
FirmF_Seafood_MAG_S013[f]	167055	1152	57	600	0.07	Zone 3	Architecture
FirmF_Seafood_MAG_S014	299	383894	355	1337197	59.42	Zone 3	Architecture
FirmF_Seafood_MAG_S073	301	547005	824	1999796	73.2	Zone 3	Equipment
FirmF_Seafood_MAG_S077	301	958444	1240	3371738	86.81	Zone 3	Architecture
FirmG_Dairy_MAG_S051[f]	167268	141	0	445	0.07	Zone 3	Architecture
FirmG_Dairy_MAG_S134	190	252961	1357355	658	69.32	Zone 3	Architecture
FirmG_Dairy_MAG_S135	63	165311	881033	512	50.03	Zone 3	Architecture
FirmG_Dairy_MAG_S169	7838	31714	151179	12588	17.96	Zone 3	Architecture
FirmG_Dairy_MAG_S170	16480	214483	918154	135648	57.59	Zone 3	Architecture
FirmI_Dairy_MAG_S064[g]	112297	3432	2359	793	0.2	Zone 3	Architecture
FirmI_Dairy_MAG_S105	33	652459	2706450	2239	95.53	Zone 3	Architecture
FirmI_Dairy_MAG_S110	555	589334	2400074	1912	91.34	Zone 2	Architecture
FirmJ_Dairy_MAG_S016	683	501055	1035	2013650	91.8	Zone 1	Equipment
FirmJ_Dairy_MAG_S074	1105	118808	171	467965	28.68	Zone 2	Equipment
FirmJ_Dairy_MAG_S078	861	170530	300	677405	41.34	Zone 2	Equipment
FirmL_Seafood_MAG_S032[e]	149837	3135	1	13641	1.32	Zone 3	Architecture
FirmL_Seafood_MAG_S097	3299	36092	0	170498	9.8	Zone 1	Equipment
FirmL_Seafood_MAG_S108	477	421926	1021	1534236	78.84	Zone 1	Equipment
FirmL_Seafood_MAG_S122	465	494474	938	1847776	79.02	Zone 3	Architecture
FirmL_Seafood_MAG_S123	467	375019	930	1384221	80.25	Zone 3	Architecture
FirmL_Seafood_MAG_S125	23160	129922	381978	185039	51.52	Zone 3	Architecture
FirmW_Seafood_MAG_S082	918	634905	1341	2454638	71.71	Zone 2	Equipment

[a]Number of missing Single Nucelotide Polymorphisms (SNPs)

[b]Reads mapped to *Listeria monocytogenes* (no lineage)

[c]Reads mapped to *Listeria monocytogenes* lineage I

[d]Reads mapped to *Listeria monocytogenes* lineage II

[e]Samples placed on the tree but outside of the cluster of the corresponding WGS isolate

[f]Samples could not be placed on the tree due to low genome coverage

[g]Sample clustered with a *Listeria innocua* reference isolate

[h]Relative Abundance (%) of *Listeria monocytogenes*

Table S4 Kmer analysis statistics of metagenomic assembled genomes (MAG) included in this study (n=33). Table includes sample name, number of missing Single Nucleotide Polymorphisms (SNP), sequencing reads mapped to *Listeria monocytogenes* (no lineage), sequencing reads mapped to *Listeria monocytogenes* lineage I and sequencing reads mapped to *Listeria monocytogenes* lineage II. Samples that could not be placed on the tree due to low genome coverage are also included (n=4).

Sample	Total Reads	*Listeria* reads	Contigs > 500bp	Largest Contig	Total Length	GC%	N50	Coverage	Missing SNPS	SRST2_results
FirmI_Dairy_MAG_S105_0.5%	26036	18136	1700	5513	1,800,083	38.6	1147	3.3	14417	Not found
FirmI_Dairy_MAG_S105_1%	52200	36238	1359	13188	2,658,850	38.3	2542	3.4	12981	Not found
FirmI_Dairy_MAG_S105_1.5%	78484	54863	730	29478	2,894,938	38.1	6415	3.8	5381	Lineage I
FirmI_Dairy_MAG_S105_2%	103359	71575	487	68299	2,982,645	38.1	10941	4.4	1471	Lineage I
FirmI_Dairy_MAG_S105_3%	156347	109065	259	92405	3,032,431	38.1	27941	6.4	146	Not found
FirmI_Dairy_MAG_S105_4%	207538	144552	169	164858	3,045,748	38.0	48914	8.4	38	Not found
FirmI_Dairy_MAG_S105_8%	416594	290711	157	306976	3,106,657	38.3	173353	16.9	0	Not found
FirmI_Dairy_MAG_S105	5196513	3623130	94	728777	3,167,015	38.3	476846	247.8	0	Not found
FirmG_Dairy_MAG_S170_2%	76257	27140	3690	10289	3,584,294	41.6	1005	3.7	30713	Not found
FirmG_Dairy_MAG_S170_3%	115644	41067	4911	12934	4,995,778	42.3	1052	4.0	20901	Not found
FirmG_Dairy_MAG_S170_4%	155204	55144	5999	19662	6,266,012	43.1	1067	4.5	11967	Not found
FirmG_Dairy_MAG_S170_6%	231976	82822	7880	23171	8,548,398	44.4	1132	5.8	5850	Not found
FirmG_Dairy_MAG_S170_8%	306870	108794	9261	33223	10,488,589	45.2	1235	6.9	3190	Lineage I
FirmG_Dairy_MAG_S170_12%	462090	163740	11552	41703	13,968,617	46.5	1406	8.2	1889	Not found
FirmG_Dairy_MAG_S170_16%	617326	219794	13127	52587	16,683,247	47.4	1553	8.9	1313	Not found
FirmG_Dairy_MAG_S170	3852107	1367591	32631	165322	49,244,213	52.6	2207	21.2	3463	Not found
FirmI_Dairy_MAG_S064_8%	509428	18853	9888	125929	13,339,666	40.9	1566	8.0	275743	Not found
FirmI_Dairy_MAG_S064_16%	1021011	37254	12482	81261	20,109,115	41.2	2370	10.4	128904	Not found
FirmI_Dairy_MAG_S064_24%	1532278	56253	13531	63049	24,129,949	41.8	3223	11.4	32041	Not found
FirmI_Dairy_MAG_S064_32%	2045787	75315	14763	137642	27,220,400	42.4	3750	12.9	9367	Not found
FirmI_Dairy_MAG_S064_48%	3066563	112339	18060	189521	32,569,387	43.4	3868	16.9	4303	Not found
FirmI_Dairy_MAG_S064_66%	4216252	154237	21467	239189	37,679,240	44.4	3273	22.1	2073	L. innocua
FirmI_Dairy_MAG_S064	6385544	234129	29394	509326	46,731,215	45.5	2234	31.2	1940	Not found

Table S5 *In silico* fastq dilutions. Sample name, total number of reads obtained by shotgun metagenomic sequencing, number of *Listeria monocytogenes* reads, total contigs greater than 500bp assembled using metaspades, largest contig, total length of the contig, GC%, N50, coverage and missing SNPs are shown for each of the fastq dilutions for the 3 shotgun metagenomic samples chosen.
